# LVIS-within-enterprise double-stent technique with coil embolization in the treatment of patients with acutely ruptured intracranial vertebrobasilar artery-dissecting aneurysms

**DOI:** 10.3389/fneur.2023.1069380

**Published:** 2023-03-24

**Authors:** Qiaowei Wu, Yuxiao Meng, Aixia Chen, Shancai Xu, Chunlei Wang, Zhiyong Ji, Jingtao Qi, Kaikun Yuan, Jiang Shao, Huaizhang Shi, Pei Wu

**Affiliations:** Department of Neurosurgery, The First Affiliated Hospital of Harbin Medical University, Harbin, Heilongjiang, China

**Keywords:** vertebrobasilar artery, dissecting aneurysms, ruptured, subarachnoid hemorrhage, stent-assisted coiling

## Abstract

**Objective:**

This study aimed to evaluate the feasibility of the low-profile visualized intraluminal support (LVIS)-within-enterprise double-stent technique for patients with acutely ruptured intracranial vertebrobasilar artery-dissecting aneurysms (ari-VBDAs).

**Methods:**

A total of 30 patients with ari-VBDAs who underwent reconstructive treatment using LVIS-within-enterprise double-stent technique with coil embolization between January 2014 and May 2022 were retrospectively enrolled. Patients' characteristics and clinical and imaging outcomes were reviewed. The functional outcomes were assessed using the modified Rankin scale (mRS).

**Results:**

A total of 34 ari-VBDAs were identified, including seven (20.6%) basilar artery aneurysms and 27 (79.4%) vertebral artery aneurysms. All aneurysms were successfully treated in the acute phase. In total, six (20.0%) patients experienced in-hospital serious adverse events, including two deaths (6.7%). The median clinical follow-up time of the remaining 28 patients was 20.0 (IQR, 7.3–40.8) months. The incidences of dependency or death (mRS score of 3–6) at discharge and at the last follow-up were 16.7% and 14.3%, respectively. Aneurysm rebleeding occurred in one (3.3%) patient periprocedurally. In total, three (10.0%) patients had ischemic events, one of which occurred during the periprocedural period and two occurred during follow-up. A total of two patients (6.7%) underwent ventriculoperitoneal shunt. Imaging follow-up was available for 14 patients at the median of 12.0 (IQR, 7.0–12.3) months, with a complete occlusion rate of 93.3% (14/15). In total, one patient experienced parent artery occlusion, and no aneurysm was recanalized.

**Conclusion:**

LVIS-within-enterprise double-stent technique with coil embolization for the treatment of patients with ari-VBDAs could be performed with a good safety profile and high technical success rate. The rate of complete aneurysm occlusion during follow-up seemed to be satisfactory.

## Introduction

The prognosis of posterior circulation aneurysmal subarachnoid hemorrhage is poor (1, 2). For patients with acutely ruptured intracranial vertebrobasilar artery-dissecting aneurysms (ari-VBDAs), if untreated, Mizutani et al. (3) reported that approximately 70% of patients subsequently underwent rebleeding, and it most commonly occurred within the first 24 h, resulting in the deaths of approximately half of those patients. Previous studies have shown that endovascular therapy tended to have better outcomes than neurosurgical clipping in the treatment of posterior circulation aneurysms (4, 5). Deconstructive treatment and single stent-assisted or multilayer stent-assisted coiling of ari-VBDAs have been described in published studies (6, 7). However, ari-VBDAs are rare, and there are limited data on the treatment outcomes regarding the imaging results and the benefits/risks of different endovascular techniques. The safety and efficacy of different endovascular treatment techniques for ari-VBDAs remain to be further explored.

**Figure 1:**
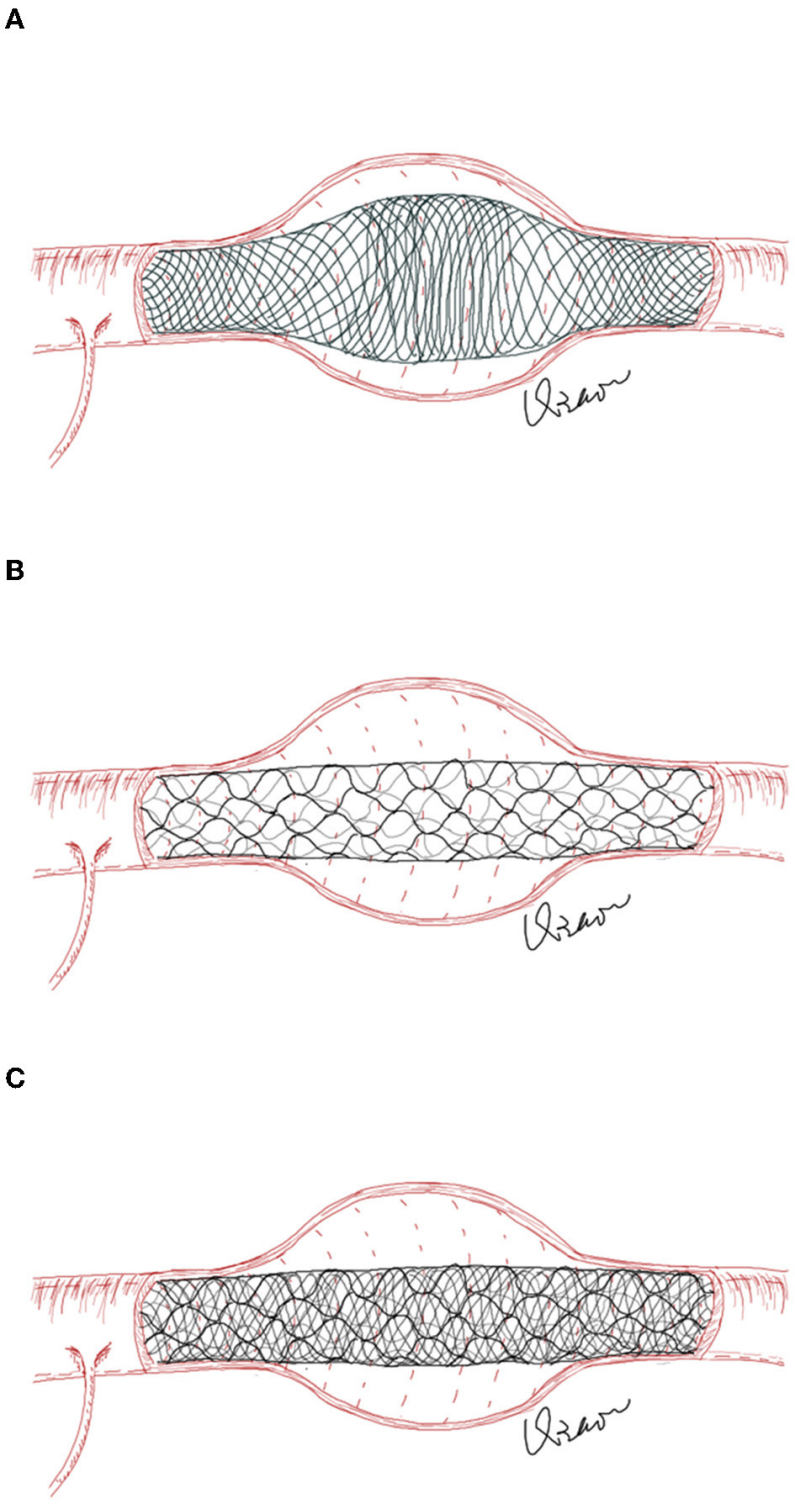
**(A)** In fusiform aneurysms, the LVIS stent will expand outward along the aneurysm neck, resulting in an uneven distribution of the stent's metal coverage along the aneurysm neck. **(B)** The enterprise stent has a greater radial force but less metal coverage than the LVIS stent. **(C)** The enterprise stent is first deployed to play the role of “the skeleton,” which could reduce the unconstrained segment size across the aneurysm neck and limit the outward expansion of the LVIS stent.

A low-profile visualized intraluminal support (LVIS) device has been demonstrated to be beneficial in assisting the coil embolization of intracranial aneurysms (8). However, due to the nature of the braided stent, the LVIS stent will expand outward along the neck when treating fusiform-dissecting aneurysms, and an uneven distribution of metal coverage along the neck may affect the flow-diverting effect (9). To avoid this shortcoming, in this study, we attempted to use LVIS-within-enterprise double-stent technique with coil embolization in the treatment of ari-VBDAs. The enterprise stent was used as an external frame to limit the expansion of the LVIS stent to increase metal coverage in the aneurysmal neck (Figure 1). The current study aimed to assess the feasibility of the LVIS-within-enterprise double-stent technique with coil embolization in the treatment of ari-VBDAs.

## Materials and methods

### Patient population

In this retrospective analysis of our single-center database, a consecutive series of patients with ari-VBDAs treated with LVIS-within-enterprise double-stent technique with coil embolization between January 2014 and May 2022 were reviewed. The inclusion criteria for patients eligible for the study were as follows: (1) patients who received endovascular treatment within 72 h after subarachnoid hemorrhage (SAH); (2) SAH was confirmed by computer tomography (CT), and target aneurysm was confirmed by digital subtraction angiography (DSA) or CT angiography; (3) fusiform-dissecting aneurysm originating from the main trunk of the vertebrobasilar artery; and (4) aneurysm treated with LVIS-within-enterprise double-stent technique with coil embolization. The exclusion criteria were as follows: (1) aneurysm involving an extracranial segment of the vertebral artery or aneurysm originating from the branch artery; (2) basilar tip aneurysm, superior cerebellar artery aneurysm, or posterior cerebral artery aneurysm; (3) complications caused by other treated cerebral aneurysms; and (4) saccular shape, vertebrobasilar dolichoectasia, traumatic, or iatrogenic aneurysms.

A total of 30 patients harboring 34 ari-VBDAs, who underwent the endovascular treatment using LVIS-within-enterprise double-stent technique with coil embolization, were included in this study. The institutional review board of the First Affiliated Hospital of Harbin Medical University approved this retrospective study, and written informed consent was obtained from all the patients before the procedure.

### Antiplatelet therapy

A loading dose of 300 mg clopidogrel and 300 mg aspirin was administrated orally or *via* a nasogastric tube at least 2 h before stenting, followed by a conventional dosage of dual antiplatelet therapy (75 mg clopidogrel + 100 mg aspirin) daily for at least 3 months. Clopidogrel was discontinued 3 months after the procedure, and aspirin was maintained indefinitely (if no contraindication). In addition, intraprocedural tirofiban administration as an alternative to preprocedural oral antiplatelet therapy was also used. Tirofiban was administered as an intravenous bolus (8 μg/kg) over a 3-min period during stenting, and a maintenance dose of 0.1 μg/kg/min was followed for at least 24–48 h after the procedure. Approximately 2 h before tirofiban was discontinued, a loading dose of 300 mg clopidogrel and 300 mg aspirin was administered orally or *via* a nasogastric tube, while the maintenance dose of tirofiban was halved, followed by a conventional daily dual antiplatelet therapy (75 mg clopidogrel + 100 mg aspirin) (10). At least 3 days after the dual antiplatelet therapy, thromboelastography (TEG) was used to monitor the platelet function of every patient. If clopidogrel hypo-responders or non-responders were detected, clopidogrel would be changed to ticagrelor 90 mg, twice daily.

### Procedures

After sheath placement, a suitable guiding catheter was placed in the distal vertebral artery. Three-dimensional (3D) rotational angiography was performed, and 3D reconstruction was used to determine the work projection, measure the parent artery and aneurysms, and determine the size of the stents. The selection of stent size was generally based on the larger proximal vessel diameter of the parent artery. If the vessel was tortuous or the aneurysm neck was very wide, an appropriately larger stent size could be selected for better anchoring. The aneurysm was first coiled with the assistance of an enterprise stent (Codman Neurovascular, Massachusetts, USA) using the jailing or semi-jailing technique. After placement of the enterprise stent, the anchoring distance between the two ends of the aneurysm should be at least 5 mm. The second LVIS stent (MicroVention-Terumo, California, USA) was then deployed within the enterprise stent. The aneurysm was coiled until satisfactory, and/or additional packing was not possible.

### Data collection

The following baseline variables were collected: patient demographics (including sex and age), clinical data [including the World Federation of Neurological Surgeons (WFNS) grade, modified Fisher grade, history of hypotension and diabetes mellitus, and smoking and alcohol abuse status], aneurysmal data (including the location, maximal diameter, and incorporation of the branch vessel), and procedural related data (including the number of coils used and procedure time).

Clinical outcomes were evaluated using the modified Rankin scale (mRS) score at discharge and at the last follow-up. An mRS score of 3–5 was regarded as a dependency, and an mRS score of 6 referred to the death of the patient. Postprocedural serious adverse events (SAEs) including rebleeding, ischemia, shunt-dependent hydrocephalus, or other threatening events, leading to hospitalizations or prolonged hospitalizations, were recorded. Ischemic events were defined as the following: (1) in-stent thrombosis, partial or complete occlusion of the proximal or distal arteries on DSA and (2) thromboembolism symptoms (excluding vasospasm) with or without corresponding cerebral infarction on magnetic resonance imaging (MRI)/CT. Hemorrhagic events were defined as follows: (1) postprocedural CT/MRI showing new intracerebral hemorrhage with or without clinical symptoms and (2) new subarachnoid hemorrhage on CT.

Technical success was defined as satisfactory coiling and stable stent placement with complete coverage of the aneurysm neck and patency of the parent artery. Imaging follow-up with DSA, CT angiography, or magnetic resonance angiography was performed at 6–12 months postoperatively. Aneurysm occlusion status and parent artery patency were evaluated.

Normally distributed continuous variables were summarized as mean ± standard deviation (SD), while non-normally distributed continuous variables were summarized as the median and interquartile range (IQR). Categorical variables were summarized as numbers followed by percentages.

## Results

### Patient demographic and baseline characteristics

A total of 30 patients harboring 34 ari-VBDAs were included in this study, with a mean age of 53.3 ± 12.7 years. The cohort comprised 18 (60.0%) male patients and 12 (40.0%) female patients. More than half of the patients had a history of hypertension (16/30, 53.3%), and nearly half had a history of smoking (40.0%, 12/30). Among the 30 patients, there were 12 patients of WFNS grade 1 (12/30, 40%), 10 patients of grade 2 (10/30, 33.3%), one patient of grade 3 (1/30, 3.3%), six patients of grade 4 (6/30, 20.0%), and one patient of grade 5 (1/30, 3.3%). In terms of the modified Fischer grade, there was one patient of grade 1(1/30, 3.3%), 15 patients of grade 2 (15/30, 50.0%), and 14 patients of grade 4 (14/30, 46.7%). Before the SAH occurred, 25 (25/30, 83.3%) patients had no symptoms (mRS score of 0), three (3/30, 10.0%) patients had mild symptoms (mRS 1), and two (2/30, 6.7%) patients had a slight disability (mRS 2). Among the 34 aneurysms, 27 (27/34, 79.4%) aneurysms originated from the vertebral artery and seven (7/34, 20.6%) aneurysms originated from the basilar artery. In total, five aneurysms (5/34, 14.7%) involved the posterior inferior cerebellar artery (PICA) or anterior inferior cerebellar artery (AICA). The median aneurysm maximum diameter was 6.6 [interquartile (IQR), 5.0–9.0] mm. The detailed characteristics are given in Table 1.

**Table 1:** Baseline characteristics of patients and aneurysms.

**Characteristics**	***n* = 30 patients (34 aneurysms)**
Male, *n* (%)	18 (60.0)
Mean age, years (±SD)	53.3 ± 12.7
**Risk factors**, ***n*** **(%)**	
Hypertension	16 (53.3)
Diabetes mellitus	4 (13.3)
Smoking	12 (40.0)
Alcohol abuse	7 (23.3)
**mRS before onset**, ***n*** **(%)**	
0	25 (83.3)
1	3 (10.0)
2	2 (6.7)
**WFNS**, ***n*** (%)^*^	
Grade 1	12 (40.0)
Grade 2	10 (33.3)
Grade 3	1 (3.3)
Grade 4	6 (20.0)
Grade 5	1 (3.3)
**Modified Fisher**, ***n*** **(%)**	
Grade 1	1 (3.3)
Grade 2	15 (50.0)
Grade 3	0
Grade 4	14 (46.7)
Median maximum diameter of aneurysm, mm (IQR)	6.6 (5.0-9.0)
**Aneurysm location**, ***n*** **(%)**	
BA	7 (20.6)
VA	27 (79.4)
Aneurysms involving side branches, *n* (%)	5 (14.7)

^*^Percentage totaling 99.9% due to rounding; mRS, modified Rankin scale score; WFNS, World Federation of Neurological Surgeons; IQR, interquartile range; BA, basilar artery; VA, vertebral artery.

### Procedural data

A total of 30 LVIS stents and 30 enterprise stents were deployed in 30 patients, with a technical success rate of 100%. In total, two patients were treated with two overlapping stent-assisted coiling for two tandem aneurysms, and one patient was treated with two overlapping stent-assisted coiling for three tandem aneurysms. The median coil usage per aneurysm was 4.0 (IQR, 3.0–6.0), and the median procedure time per patient was 102.5 (IQR, 60.0–108.8) min. External ventricular drainage was performed in one patient.

### In-hospital SAEs

There were six (6/30, 20.0%) patients who experienced in-hospital SAEs, including two deaths (6.7%). In total, one patient with the admission WFNS grade of 2 experienced rebleeding the day after the procedure and subsequently died. One patient with the admission WFNS grade of 5 died due to the severity of the initial aneurysm rupture. One patient experienced unilateral limb weakness 1 day after the procedure, and the mRS at discharge was 3. One patient with the admission WFNS grade of 1 developed hydrocephalus, and the ventriculoperitoneal shunt was then performed (mRS at discharge was 4). Two patients had pneumonia.

### Clinical outcomes

The incidence of dependency or death (mRS score of 3–6) at discharge was 16.7% (5/30). The median clinical follow-up time of the remaining 28 patients was 20.0 (IQR, 7.3–40.8) months. In total, four (14.3%, 4/28) patients developed new symptoms during follow-up, and the incidence of dependency or death (mRS score of 3–6) at the last follow-up was 14.3% (4/28). During the follow-up, one patient died of intracerebral hemorrhage, and one patient died of acute cerebral infarction; one patient developed hydrocephalus, a ventriculoperitoneal shunt was then performed, and the mRS score at the last follow-up was 0; one patient experienced unilateral limb weakness, and the mRS score at the last follow-up was 1. Outcome details are shown in Table 2.

**Table 2:** Outcome details.

**Details**	**Number of patients**
In-hospital SAEs^*^^†^, ***n*** **(%)**	
Rebleeding	1 (3.3)
Initial aneurysm rupture-related death	1 (3.3)
Ischemia	1 (3.3)
Shunt-dependent hydrocephalus	1 (3.3)
Pneumonia	2 (6.7)
**mRS at discharge**^†^, ***n*** **(%)**	
0–2	25 (83.3)
3–6	5 (16.7)
**Follow-up symptoms**^‡^, ***n*** **(%)**	
Intracerebral hemorrhage	1 (3.6)
Ischemia	2 (7.1)
Shunt-dependent hydrocephalus	1 (3.6)
**mRS at last follow-up**^‡^, ***n*** **(%)**	
0–2	24 (85.7)
3–6	4 (14.3)
**Occlusion status**^**§**^, ***n*** **(%)**	
Completely occluded	14 (93.3)
Incompletely occluded	1 (6.7)

^*^Percentage totaling 99.9% due to rounding; SAEs, serious adverse events; mRS, modified Rankin scale;^†^*n* = 30;^‡^*n* = 28; ^§^*n* = 15.

### Imaging outcomes

**Figure 2:**
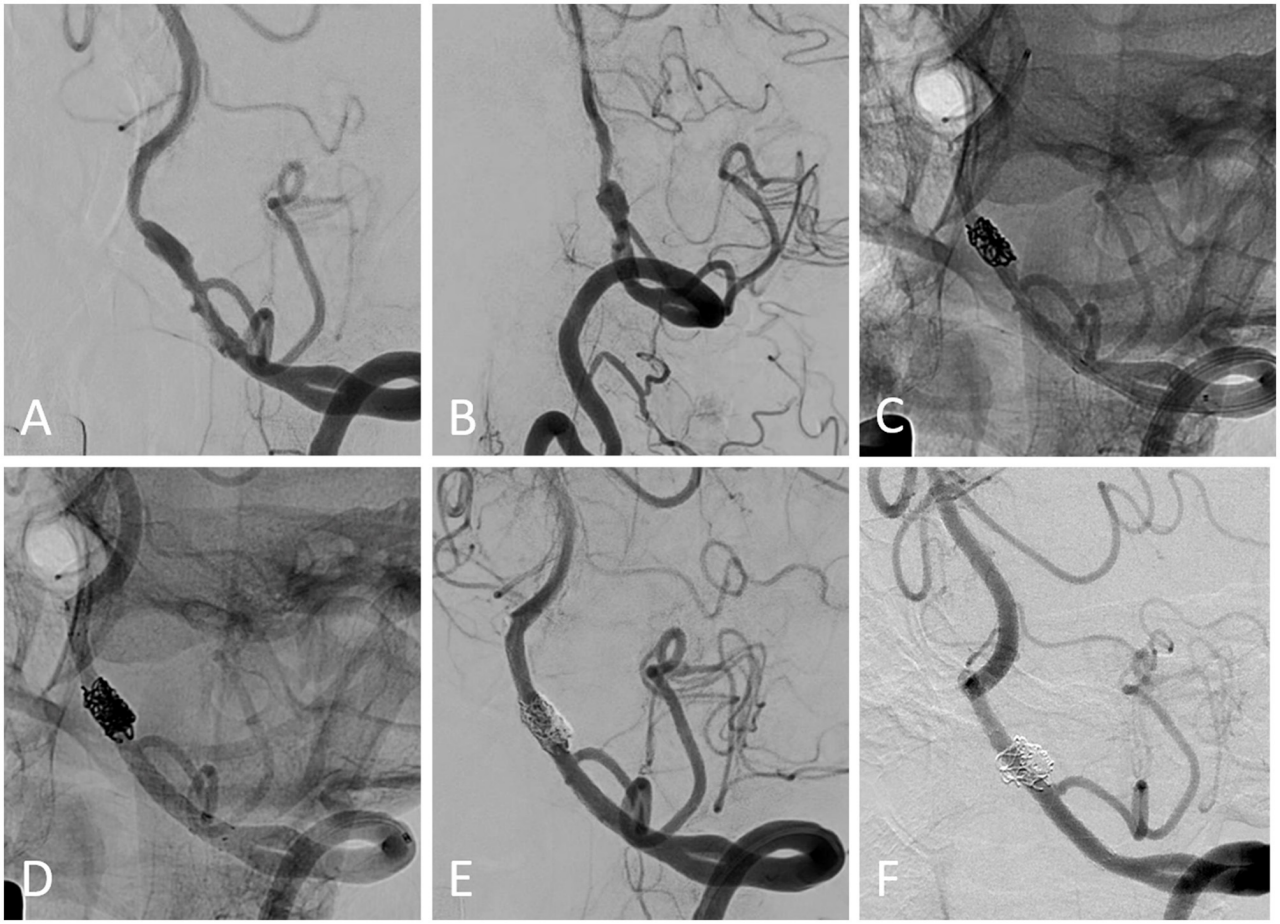
**(A, B)** Pretreatment digital subtraction angiography (DSA) from a patient harboring a left-sided vertebral artery-dissecting aneurysm. **(C)** The first enterprise stent was deployed after partial coil embolization using the semi-jailing technique. **(D)** The following LVIS stent was deployed within the enterprise stent, and additional coiling was performed. **(E)** The immediate postprocedural DSA showed the coiling result. **(F)** Angiographic follow-up showed the complete occlusion of the aneurysm and patency of the parent artery.

Imaging follow-up was available for 14 patients with 15 aneurysms at the median of 12.0 (IQR, 7.0–12.3) months, with a complete occlusion rate of 93.3% (14/15) (Figure 2). In total, one patient presented with a residual aneurysm sac after the embolization, and the aneurysm remained unchanged at 12 months of imaging follow-up. One patient experienced unilateral limb weakness 3 months after the procedure, and subsequent DSA indicated occlusion of the parent artery. After intensive antiplatelet therapy, the patient's symptoms were relieved without surgical intervention.

## Discussion

Despite the variety of acute treatment approaches for patients with ari-VBDAs, the prevailing view is that early intervention reduces mortality and leads to favorable clinical outcomes (2, 3). However, the technique for treating wide-necked aneurysms should be chosen carefully. The deconstruction technique for vertebrobasilar dissecting aneurysms (VBDAs) has been shown to be associated with higher occlusion rates in published studies (11, 12). Due to the pathological features of the vertebrobasilar artery and its relationship to perforated branches, the indications of the deconstruction technique are strictly limited, and it is only suitable for patients with good collateral vascularization. Reconstructive treatment including flow diversion (FD), single stent-assisted coiling, and conventional series stent-assisted coiling is the mainstream treatment for VBDAs (6, 7, 13, 14). A meta-analysis by Domingo et al. (15) found similar efficacy in the occlusion rate for posterior circulation non-saccular aneurysms treated with conventional stent-assisted coiling and FD, with the complete/near-complete occlusion rates of 84% and 83%, respectively. However, due to antiplatelet regimens, device prices, and insurance coverage, the use of FD in treating ari-VBDAs has certain limitations. In addition, a meta-analysis suggests that fusiform and dissecting aneurysms may be one of the risk factors for complications of intracranial aneurysms treatment with FD (16). The off-label use of FD for the treatment of ari-VBDAs still needs further confirmation. Conventional single stent-assisted coiling, to some degree, was associated with risks of aneurysm recurrence (7, 13).

Low-profile visualized intraluminal support is a self-expanding, retrievable, braided intracranial stent indicated for the treatment of wide-necked intracranial aneurysms with ~ 23% metal coverage, and previous studies have demonstrated favorable safety and efficacy profiles (8, 17). Tian et al. (18) compared the hemodynamic effect of the pipeline flow diverter and LVIS stent in aneurysm models and found that a compacted LVIS stent may provide a flow diversion effect comparable to that of PED. However, due to the braided design, in fusiform aneurysms, the LVIS stent will expand outward along the aneurysm neck, resulting in an uneven distribution of the stent's metal coverage along the aneurysm neck, which may affect the flow-diverting effect and aneurysm-healing process. Matsuda et al. (9) deployed an LVIS blue stent in a fusiform aneurysm model and found that there were three zones of different metal coverages along the stent, defined as the mid-zone, the transition zone, and the high-density zone. The transition zone was defined as the transitional portion of the aneurysm neck and parent artery, which had the lowest metal coverage. In addition, the outward expansion may also cause proximal or distal shortening of the stent, increasing the risk of stent malposition or migration. An enterprise stent has greater radial force but less metal coverage (~ 8%) than the LVIS stent. As a laser-cut, closed cell stent, the radial force of the enterprise stent is greater than that of the LVS stent. The enterprise stent is first deployed to play the role of “the skeleton,” which could reduce the unconstrained segment size across the aneurysm neck and limit the outward expansion of the LVIS stent. The LVIS-within-enterprise stenting could maintain the flow-diverting effect with a relatively high metal coverage distribution and promote the aneurysm healing process. Moreover, the unconstrained length of the LVIS stent ranges from 10 to 30 mm, and due to the braided design, the LVIS stent is at risk of shortening and migrating, which may be insufficient to cover aneurysms with long-segment lesions. The length of the enterprise stent ranges from 14 mm to 37 mm, and the original length can be maintained after deployment. Therefore, in some patients with lengthy lesions, the LVIS-within-enterprise stenting not only provides better flow-diverting effects but also prevents stent shortening or migrating.

As reported by Mizutani et al. (3), for patients with ari-VBDAs, 70% of the patients underwent rebleeding, and 56.7% of the rebleeding occurred within 24 h and 80% occurred within the 1st week, resulting in a mortality rate of 46.7%. To reduce the rate of rebleeding, early intervention to completely occlude the ruptured aneurysm is necessary. However, many cases of recurrent VBDAs after endovascular treatment have been reported (7, 13, 19). In their study, Kim et al. (13) found that the recurrence rate was 19.4% for patients with vertebrobasilar fusiform aneurysms, with a mean follow-up time of 9.2 months. The other study by Kim et al. (19) showed a recurrence rate of 13% for patients with ruptured or unruptured VBDAs and found that PICA involvement was associated with recurrence. In our study, we attempted to use LVIS-within-enterprise stent-assisted coiling in the treatment of patients with ari-VBDAs and demonstrated a complete occlusion rate of 93.3%. No aneurysm recurred in the available imaging follow-up data.

A retrospective study by Church et al. (20) included 84 ruptured or unruptured posterior circulation fusiform aneurysms treated with microsurgical and endovascular approaches. The authors reported that the neurological complication rate was 14%, and 67% of the complications were ischemic strokes. Peng et al. (21) noted that the procedure-related complication was 23.4% for patients with basilar trunk and vertebrobasilar junction aneurysms, including 16.9% of patients with ischemic complications. Another study reported that the overall SAEs rate and ischemic stroke rate were 15.7% and 13.7%, respectively, after the endovascular treatment of vertebrobasilar aneurysms (6). Published studies have reported that ischemic complications accounted for the majority of all complications after the endovascular treatment of posterior circulation or vertebrobasilar aneurysms. In our study, the incidences of periprocedural and overall postprocedural ischemic complications were 3.3% and 10.0%, respectively, which were similar to previous findings (6, 20, 21). Inadequate stent expansion, antiplatelet drug hyporesponse or non-response, and thrombus detachment during the stenting or coiling may be the potential causes of ischemic complications after endovascular therapy.

Another problem with the endovascular treatment of a ruptured aneurysm is rebleeding. In the ISAT trial, the rebleeding rate was 4.2% during the 1st year after endovascular treatment, and a meta-analysis of 2,121 patients conducted by Boogaarts et al. (22) revealed that a large aneurysm size is associated with aneurysmal rebleeding. In our study, one patient experienced postprocedural rebleeding (aneurysm maximum diameter: 15.6 mm). The coil embolization and deployment of relatively high metal coverage stents may lead to rapid intra-aneurysmal thrombus formations. The autolysis of the aneurysm wall associated with acute intra-aneurysmal thrombosis may be a possible cause of rebleeding (23).

There are some limitations. The present study was retrospectively designed with a relatively small sample size, and the potential bias inherent in retrospective research was inevitable. In addition, among the surviving patients, 46.2% of patients were lost to imaging follow-up, which might bias the evolution of the aneurysm occlusion. Moreover, the present study was not compared with other treatment modalities, and the differences in safety and efficacy between LVIS-within-enterprise stenting and other treatment modalities are unclear. Thus, further comparative studies with long-term follow-up are needed.

## Conclusion

The findings in this study suggest that the LVIS-within-enterprise double-stent technique with coil embolization may be a feasible method for the treatment of ari-VBDAs, and have a good safety profile and high technical success rate. The rate of complete aneurysm occlusion during follow-up seemed to be satisfactory.

## Data availability statement

The raw data supporting the conclusions of this article will be made available by the authors, without undue reservation.

## Ethics statement

The studies involving human participants were reviewed and approved by the Institutional Review Board of The First Affiliated Hospital of Harbin Medical University. The patients/participants provided their written informed consent to participate in this study. Written informed consent was obtained from the individual(s) for the publication of any potentially identifiable images or data included in this article.

## Author contributions

QW, YM, HS, and PW contributed to the study conception and design. QW, YM, AC, SX, CW, ZJ, JQ, KY, and JS contributed to data acquisition, data interpretation, and data analysis. QW and YM drafted the manuscript. HS and PW contributed to the major revision of the manuscript. HS, PW, SX, CW, and ZJ contributed to the significant intellectual content. All authors made significant contributions to the study and manuscript preparation and critically revised the article and approved the final version of the manuscript.
